# Data supporting the physico-chemical characterization, cellular uptake and cytotoxicity of lipid nanocapsules

**DOI:** 10.1016/j.dib.2015.06.002

**Published:** 2015-06-17

**Authors:** P. Sánchez-Moreno, J.L. Ortega-Vinuesa, H. Boulaiz, J.A. Marchal, J.M. Peula-García

**Affiliations:** a European Center for Nanomedicine (CEN), Laboratory of Nanostructured Fluorinated Materials (NFMLab), Department of Chemistry, Materials, and Chemical Engineering “Giulio Natta”, Politecnico di Milano, 20131 Italy; b Biocolloid and Fluid Physics Group, Department of Applied Physics, University of Granada, 18071 Granada, Spain; c Biopathology and Regenerative Medicine Institute (IBIMER), Centre for Biomedical Research, University of Granada, Granada E-18100, Spain; d Department of Human Anatomy and Embryology, Biosanitary Institute of Granada (ibs.GRANADA), University Hospitals of Granada-University of Granada, Granada E-18012, Spain; e Department of Applied Physics II, University of Málaga, 29071 Málaga, Spain

## Abstract

The aim of this data article is to provide data for a basic knowledge of the properties of lipid nanocapsules, a new colloidal system with very promising applications in drug delivery. Firstly, we pay attention on how it is possible to determine their surface composition by means of electrokinetics measurements. On the other hand, we provide experimental evidences for a better understanding of the factors that determine the interactions of these nanoparticles with cells as a necessary step to guide the design of the most effective formulations. Additionally, we supply information about encapsulation efficiency of docetaxel, a potent chemotherapy drug, inside nanocapsules supporting the experimental cytotoxicity results of these nanosystems.

## 1 Specifications table

**Table:** 

Subject area	*Materials Science, Physics, Biology*
More specific subject area	*Nanotechnology, Development of drug delivery nanosystems*
Type of data	*Text and graphs*
How data was acquired	*Dynamic light-scattering. Flow cytometry. High performance liquid chromatography (HPLC).*
Data format	*Analyzed*
Experimental factors	*Lipid nanocapsules obtained by solvent displacement technique with a core of olive oil and a shell composed by lecithin, poloxamers or their mixtures.*
	*The human lung-cancer cell line (A549) and the human leukemic monocytic lymphoma cell line (U937)*
Experimental features	*Electrophoretic mobility measurements. Uptake analysis of Coumarin 6-loaded nanocapsules. Determination of the encapsulation efficiency of Docetaxel.*
Data source location	*Dpt. of Applied Physics, Faculty of Science, University of Granada, Granada, Spain.*
Data accessibility	*Data are available within this article and are related to*[1]

## 2 Value of the data

• Electrophoretic mobility data of different lipidic nanocapsules with different surface composition allow the determination of the shell composition of these nanosystems.
• Data from experiments of cell uptake using two different cell lines in both serum free and complete medium conditions, will let other researchers to know that the nature of interactions of the same material in the presence or absence of proteins differs.
• The cytotoxicity experiments show how the dosage of docetaxel necessary to induce the same cytotoxic effect in cancer cells that free docetaxel is much lesser in loaded-nanocapsules.

## 3 Data, experimental design, materials and methods

In the following sections we will present a detailed description about how to synthesize some lipidic nanocapsules with potential applications as antitumor-drug delivery carriers. As demonstrated, it is possible to get valuable information about the nature of their surfaces by means of electrophoretic mobility measurements. Subsequently, once these particles are incubated with different cancer-cell lines in presence or absence of proteins, it is demonstrated a correlation between different cellular uptake patterns observed and the different nature existing in the particle surfaces. Finally, an analysis of the drug (docetaxel) release combined with a study of cytotoxicity is also shown.

### 3.1 Material preparation

Different lipidic nanocapsule systems with the same olive oil core and shells of different composition and different surface properties were prepared using two surfactants with a markedly different nature, a non-ionic poloxamer and a charged phospholipid. The synthesis procedure was based on an emulsion solvent extraction technique previously reported [1,2]. In this way, the shell of the **EP** nanocapsules was exclusively composed by phospholipids (lecithin); in the case of the **ME** nanocapsules was composed by a mixture of lecithin and poloxamer with a predominance of the first one; for the **MP** nanocapsules both surfactants were present in the shell with a predominance of poloxamer; and finally, the **PL** nanocapsules with the shell exclusively composed by poloxamer. Docetaxel-loaded lipid nanocapsules were formulated in the same way by dissolving docetaxel in the olive oil phase at a concentration of 0.1% (w/w), while Coumarin 6 lipid nanocapsules were formulated dissolving the dye in the olive oil phase at a concentration of 0.025% (w/w).

### 3.2 Data on electrokinetic characterization

**Fig. 1:**
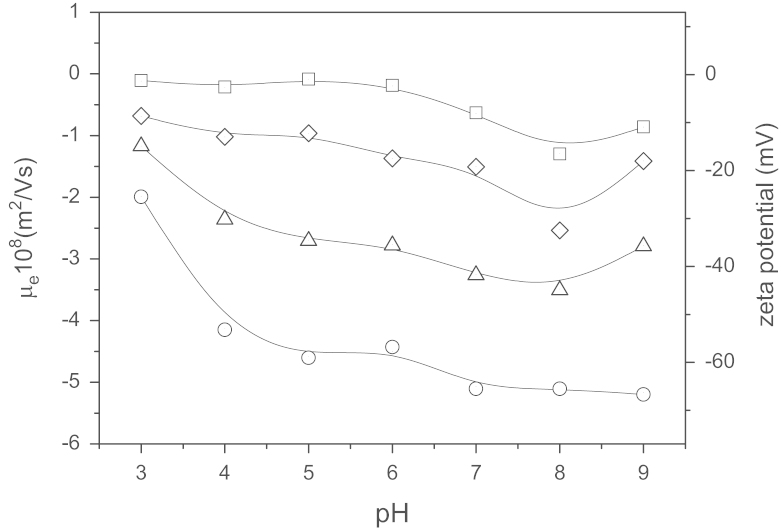
Electrophoretic mobility vs. pH in buffered media of low salinity (ionic strength equal to 0.002 M) for different nanosystems. (**○**) **EP** nanocapsules exclusively composed by lecithin (2.22 mg/ml); (Δ) **ME** nanocapsules composed by a mixture of lecithin (2.22 mg/ml) and poloxamer (0.39 mg/ml) with a predominance of the first one; (◊) **MP** nanocapsules with both surfactants, lecithin (0.22 mg/ml) and poloxamer (3.89 mg/ml) with a predominance of this last; and (□) **PL** nanocapsules, exclusively composed by poloxamer (3.89 mg/ml). Values in mg/ml refer to the amount of surfactant added in the synthesis procedure.

Electrophoretic mobility, μ_e_, was measured by Dynamic light-scattering (Zeta-Sizer NanoZ, Malvern Instruments, UK) after pouring a small volume of the nanocapsule stock (with a total surface area equal to 0.05 m^2^) into 1 mL of a low salinity solution (0.002 M) containing the buffer with the desired pH value ranging from 3 to 9. *μ*_*e*_ is an electrokinetic experimental parameter dependent of the surface electrical potential, which is ultimately governed by the composition of the lipid nanocapsules surface and by the salinity and pH conditions of the medium in which the particles are dispersed (Fig. 1).

### 3.3 Data on cellular uptake under different conditions

The U937 human leukemic monocytic lymphoma cell line (1.5×10^5^ cells), previously activated to macrophages with phorbol 12-myristate 13-acetate (PMA) for 48 h, and the A549 human lung-cancer cell line (1.5×10^5^ cells) were seeded into 6-well plates in complete culture medium (cDMEM). After 24 h, before exposure to nanoparticles, the cDMEM was removed; then, cells were washed once with PBS buffer prior to the addition of the nanoparticle dispersions. Coumarin 6-loaded nanocapsule dispersions were prepared by diluting the nanoparticle stock to the required concentration in SF or cDMEM just before addition to cells. In the control groups, cells were treated with non-fluorescent nanocarriers. After cell incubation, cells were washed three times with phosphate-buffered saline (PBS) to remove free nanocapsules, then harvested using PBS-ethylenediamine-tetraacetic acid (PBS-EDTA) and pelleted by centrifugation. Then, they were fixed at room temperature with a 4% formalin solution for 20 min and re-suspended in PBS before measuring the cell-associated fluorescence (15,000 cells per sample) using a FacsCalibur flow cytometry device (Becton Dickinson). The results are represented by averaging the distribution of cell fluorescence intensity, working with three independent replicates. Error bars represent the standard deviation between replicates. Each experiment was performed at least three times.

**Fig. 2:**
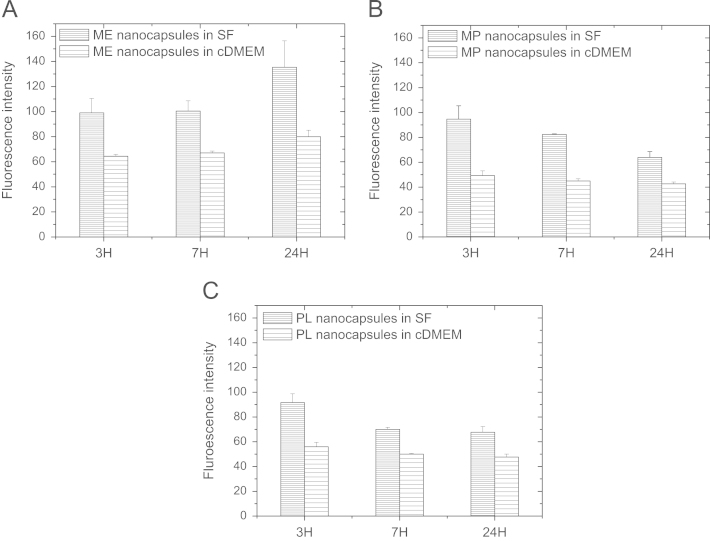
Uptake of ME (A), MP (B) and PL (C) lipid nanocapsules in SF (horizontal gray lines) and cDMEM medium (horizontal lines) by A549 cells as determined by flow cytometry. Error bars are the standard deviation of the mean cell fluorescence intensity averaged over 3 replicates. For all the types of nanocapsules, the uptake in SF was higher than in complete medium. Cells exposed to several types of particles for up to 24 h, changed their phenotype and lost cell adhesion, which is indicative of cell damage, due to the strong adhesion of bare nanoparticle surfaces to the cell membrane. In fact, we could not determine the uptake by flow cytometry of EP nanoparticles in SF because they seriously damaged cells already after short exposure times.

**Fig. 3:**
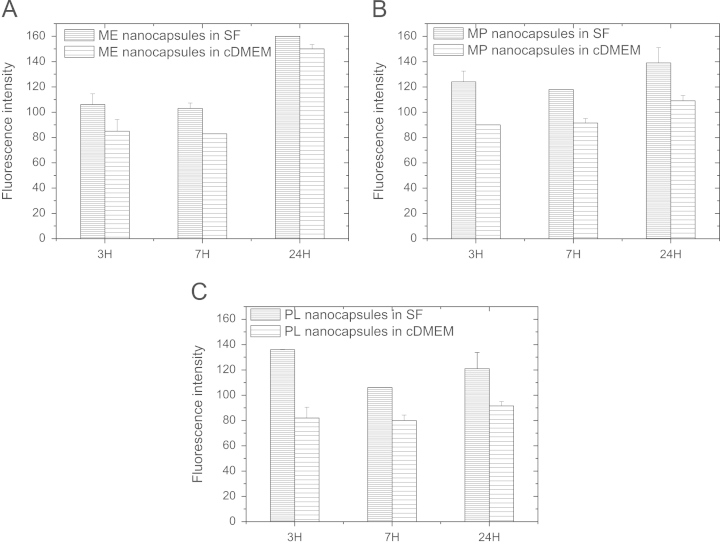
Uptake of ME (A), MP (B) and PL (C) lipid nanocapsules in SF (horizontal gray lines) and cDMEM medium (horizontal lines) by macrophages as determined by flow cytometry. For all the types of nanocapsules, the uptake in SF was higher than in complete medium.

These experiments determined the effect of the surface properties of different nanocapsules systems on the interaction with the A549 human lung-cancer cell line (Fig. 2) and the U937 human leukemic monocytic lymphoma cell line (Fig. 3) in serum free (SF) and complete medium (cDMEM) conditions.

### 3.4 Data on encapsulation of docetaxel and cytotoxicity

**Fig. 4:**
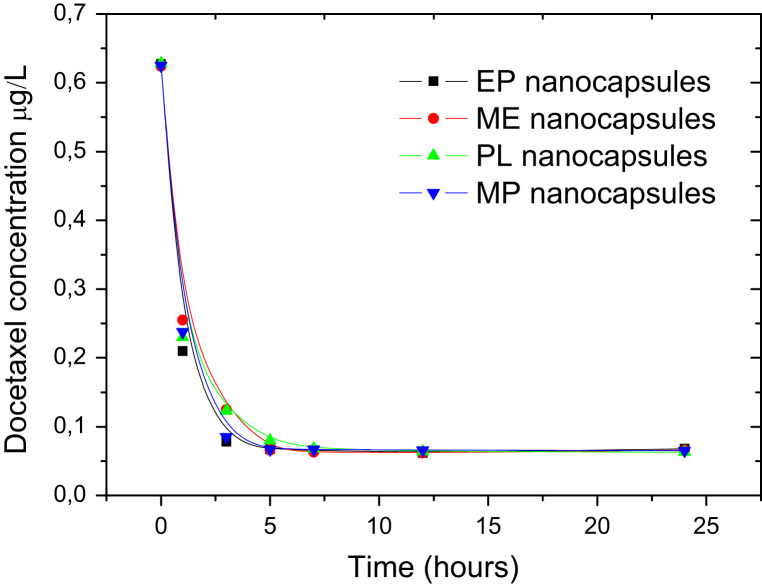
Concentration of docetaxel encapsulated in the four different nanocapsules after 1, 3, 5, 12 and 24 h of dialysis in water to remove the unbound surfactant molecules. After five hours of dialysis the 20% of docetaxel added in the synthesis remained in the oily core.

**Fig. 5:**
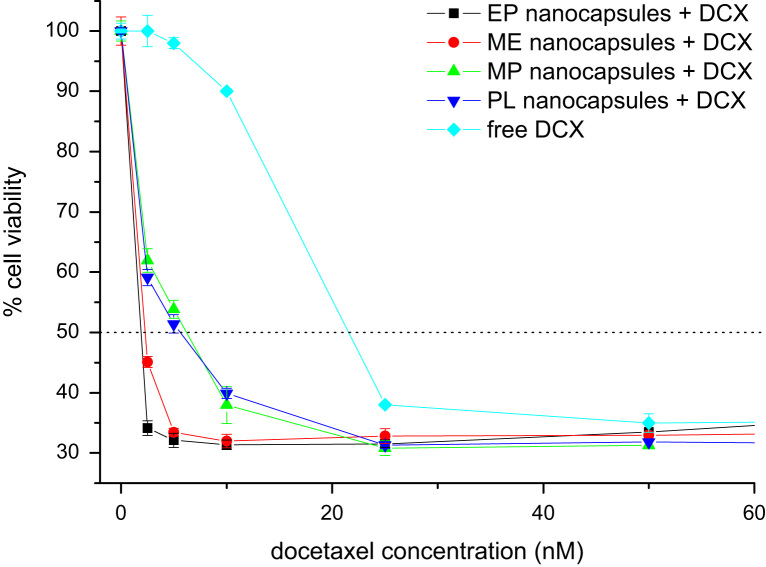
Cytotoxicity curves at different concentrations of free docetaxel and docetaxel loaded nanocapsules. Curves show that all docetaxel loaded-nanoparticles significantly decreased cell viability at low concentrations in comparison to cells treated with free docetaxel. These results indicate that the treatment with docetaxel-loaded nanocapsules allow a substantial decrease in the dosage of the drug.

The docetaxel encapsulation efficiency was calculated by HPLC at the Scientific Instrumentation Center of the University of Granada, using a SHIMADZU LC-20AC chromatograph with SPD-M20A diode array detector and C8 Nova-Pak Cartridge column (4 μm, 4.6×150 mm). Detection was performed at a wavelength of 230 nm (Fig. 4). Finally, to obtain the cytotoxicity curves, A549 cells (2×10^4^) were plated into 24-well plates with fresh cDMEM and increased concentrations of free and encapsulated docetaxel for 48 h. Then, cells were counted using the sulforhodamine-B (SRB) colorimetric assay in a Titertek Multiscan apparatus (Flow, Irvine, CA, USA) at 492 nm (Fig. 5).
